# Match running performance is influenced by possession and team formation in an English Premier League team

**DOI:** 10.5114/biolsport.2024.135414

**Published:** 2024-02-12

**Authors:** Ryland Morgans, John Radnor, Jose Fonseca, Chris Haslam, Matthew King, Dave Rhodes, Piotr Żmijewski, Rafael Oliveira

**Affiliations:** 1 School of Sport and Health Sciences, Cardiff Metropolitan University, Cardiff, UK; 2 Faculty of Human Kinetics, Lisbon University, Lisbon, Portugal; 3 Brentford FC Football Research Centre, Brentford FC, London, UK; 4 Football Performance Hub, School of Sport and Health Sciences, University of Central Lancashire, Preston, United Kingdom; 5 Jozef Pilsudski University of Physical Education in Warsaw, 00-809 Warsaw, Poland; 6 Research and Development Center Legia Lab, Legia Warszawa, Poland; 7 Research Centre in Sports Sciences, Health and Human Development, 5001–801 Vila Real, Portugal; 8 Sports Science School of Rio Maior – Instituto Politecnico de Santarem, 2040–413 Rio Maior, Santarém District, Santarém, Portugal; 9 Life Quality Research Centre, 2040–413 Rio Maior, Portugal

**Keywords:** Match running performance, Possession, Team formation, Positional demands, Soccer

## Abstract

The aim of this study was to examine the possession (very low, low, high, and very high), team formation (3-5-2 and 4-3-3) and position (centre-backs, full-backs, centre midfielders, attacking midfielders, and centre forwards) on match load across two consecutive seasons in elite soccer. Twenty-seven English Premier League outfield players were recruited. Data was monitored through an 18 Hz Global Positioning System and a 25 Hz semi-automated camera tracking system, respectively, and all variables were analysed per minute. Main effects for formation on total distance (TD) (p = 0.006; *η*^2^ = 0.010), high-speed running (HSR) (p = 0.009; *η*^2^ = 0.009), number of high metabolic load (HML) efforts (p = 0.004; *η*^2^ = 0.011) were observed. In addition, there were significant interaction effects with formation × possession on TD (p < 0.001; *η*^2^ = 0.043), HSR (p = 0.006; *η*^2^ = 0.018), sprinting (p < 0.001; *η*^2^ = 0.030), HML efforts (p < 0.001; *η*^2^ = 0.035), accelerations (p < 0.001; *η*^2^ = 0.025). From the position-specific analysis, only the running performance of centre-backs was affected by formation or positional factors. These results indicate that formation and possession can have a significant impact on TD, HSR, and HML distance. Furthermore, players performed more high-intensity efforts in 3-5-2 than 4-3-3 formation. These findings suggest that coaches can evaluate running performance in the context of formation and possession and tailor tactical strategies to optimise physical performance.

## INTRODUCTION

Soccer is a team sport known for its significant physical, technical, and tactical demands. It involves frequent intermittent bouts of high-intensity actions such as accelerations, decelerations, changes of direction, sprinting, and jumping, that are interspersed with phases of prolonged low-intensity aerobic activity, such as standing, walking and jogging [1]. Recent studies have highlighted the increasing intensity of soccer match-play over time, and assuming a similar future trend, match speed is expected to increase by ~7% by 2030 [2, 3]. Furthermore, high-intensity actions such as sprints, accelerations and decelerations are associated with the crucial moments of match-play, such as goal scoring, assisting, and defensive scenarios [4–6].

Since soccer involves the interaction of physical, technical and tactical activities among players, the adoption of differing technical/tactical strategies will result in distinct physical demands [7]. Recently, time spent in ball possession has provided a good indication of playing style. Wang et al. [8] examined the relationship between ball possession strategies, match-running performance and team success. The study found that ball possession percentage alone is not a good indicator of successful match outcome. Instead, possession affects passing (frequency and success rate), organizational aspects and running distance during ball possession. Furthermore, different ball possession strategies and durations were observed across different leagues, with the English Premier League (EPL) teams favouring a more direct playing style, while La Liga teams employed a passing through-the-pitch attack.

Additionally, during the 2018 FIFA World Cup, the majority of teams that qualified for the knockout stage adopted a higher possession-based playing style [9]. Specifically, while no significant difference in total distance (TD) was observed, teams with greater possession percentages covered more sprinting (above 25 km/h) and high-speed running (HSR) distance (between 20–25 km/h) than teams with direct-play characteristics. More recent studies have shown that teams who have finished higher in the final ranking position in La Liga and the German Bundesliga exhibited lower TD and HSR distance without ball possession, while displaying higher running outputs with ball possession [7, 10]. These results contrast with those provided by Lorenzo-Martinez et al. [11], who reported that, independent of playing position, players in very high possession teams covered significantly less distance across all speed zones.

The integration of physical and tactical demands has gained some relevance over the last few years, where an increased interest in contextualising the physical performance of soccer players concerning tactical activities [12, 13] has been observed. This novel approach has demonstrated the existence of different physical-tactical match profiles depending on playing position, both in and out of possession. It was also suggested that the adoption of generalist positions is less sensitive to estimating player’s actual physical and tactical match demands. Furthermore, it is widely accepted that the physical demands of soccer are multifactorial, and these demands may vary depending on the tactical formation, opponent standard and other contextual factors [13–18].

In this sense, tactical formation is another variable analysed recently [19]. These authors compared the three best Spanish soccer teams examining running measures with and without possession. Specifically, the first team applied a 4-4-2 formation with a compact defense and direct attack strategy, the second team applied a 4-3-3 formation with an indirect style of play and the third team applied a 4-3-3 formation with intricate attacks and effective counterattacks. These authors also analysed playing position in the different formations. The main findings reported minimal differences between the three teams. However, when considering the various positions, it was clear that team formation and the differing tactical demands have a significant influence on running performance [19]. It is also unclear whether these factors are significant in other leagues, including the EPL and thus warrant further examination.

Therefore, the aim of this study was to examine the effect of possession, team formation and playing position on physical match performance variables in an EPL soccer team across two consecutive seasons. Based on previous research [19], the study hypothesis was that possession and team formation would affect positional match running demands.

## MATERIALS AND METHODS

### Study design

This investigation utilised a two-year longitudinal study design to examine a male professional team. The team competed in the EPL during the study period that consisted of 38 official matches, 19 home and 19 away in each season. The 3-5-2 formation was utilised on 44 occasions across both study seasons, while the 4-3-3 was employed in 32 matches.

A non-probabilistic sampling protocol was employed to recruit the participants. The emphasis of the study was on monitoring player match load and the effect of possession and team formation during competitive matches. During the observation period of seasons 2021/22 and 2022/23, consistent player monitoring approaches were implemented without any interference from the researchers [20].

### Participants

Twenty-seven professional outfield first-team soccer players (age 23.2 ± 5.9 years, weight 75.2 ± 8.1 kg, height 1.83 ± 0.06 m) from an EPL club were involved in the study. Data from the complete 2021/22 and 2022/23 seasons were included in the analysis. The research inclusion criteria have been previously applied [13] and were: (i) named in the first-team squad at the start of both seasons, (ii) played in at least 80% of matches, and (iii) only completed official team training during the study period. Additionally, the exclusion criteria for the study have also been previously employed [13] and included: (i) long-term injured player data, (ii) joining the team late in either of the study seasons, (iii) lack of full, complete match data, (iv) an in-sufficient number of satellite connection signals, and (v) goalkeepers, due to the different variations in the physical demands with outfield players [21].

Players were classified into one of five positions due to varying match demands. These were: centre-backs (CB; n = 7), full-backs (FB; n = 4), centre midfielders (CM; n = 9), attacking midfielders (AM; n = 5), and centre forwards (CF; n = 2). All data collected resulted from normal analytical procedures regarding player monitoring over the competitive season [13], nevertheless, written informed consent was obtained from all participants. The study was conducted according to the requirements of the Declaration of Helsinki and was approved by the local Ethics Committee of University of Central Lancashire and the club from which the participants volunteered [22]. To ensure confidentiality, all data were anonymised prior to analysis.

### Procedure

In each season, only data from home and away EPL matches were included in the analysis. Participant data were only included in the analyses when time spent on the field exceeded 75-minutes of the match [23, 24]. For each season, players were considered when the playing time inclusion criterion was met in eight (10.5%) or more league matches across the two-season examined period. Only players with match data from both examined seasons were included in the sample. The participants performed in a median of 32% (range = 9 to 88%) of league matches across both seasons. A total of 714 individual match data points were examined across both seasons, with a median of 24 matches per player (range = 7 to 67). No data from international camps (training or matches) was included. Although, some players (n = 14) participated in 93 national team matches over both seasons (range = 2 to 8 in season 2021/22 and range = 1 to 8 in season 2022/23), this data was not utilised in the current analysis to avoid any bias when interpreting the present study results due to varying team formation and possession characteristics of the respective national teams.

### Data collection

Physical match data were consistently monitored across the study seasons using an 18 Hz Global Positioning System (GPS) (Apex Pod, version 4.03, 50 g, 88 × 33 mm; Statsports; Northern Ireland, UK) that has been previously validated for tracking distance covered and peak velocity during simulated team sports and linear sprinting [25]. All data collection procedures and unit error and reliability have previously been reported [25–28]. The distance biases for the Apex 18 Hz during a 400 m trial (1.17 ± 0.73%), 128.5 m circuit (2.11 ± 1.06%), and 20 m trial (1.15 ± 1.23%) have previously been reported [25], where these units reported a small error of around 1–2% compared to criterion distances and good levels of accuracy (bias < 5%) in sport specific metrics [25].

Following every match, running data were extracted using proprietary software (Apex, version 4.3.8, Statsports Software; Northern Ireland, UK) as software-derived data is a more simple and efficient way for practitioners to obtain data in an applied environment, with no differences reported between processing methods (software-derived to raw processed) [29]. The minimum effort duration (dwell time) to quantify HSR (0.5 s) and sprint distance (1 s) have previously been suggested [29]. Additionally, to display a higher level of precision and less error, the GPS internal processing utilised the Doppler shift method to calculate both distance and velocity [30].

Physical variables analysed were selected based on previous publications [31–33] and in practical settings are commonly utilised by elite soccer coaches. The absolute TD covered (m); HSR distance (m; distance covered > 5.5 m/s); sprint distance (m; distance covered > 7 m/s); high metabolic load distance (HMLD) (m; the total amount of HSR, coupled with the total distance of accelerations (> 3 m/s^2^) and decelerations (< -3 m/s^2^). This variable refers to the distance covered with a power consumption above 25.5 W/kg. This value corresponds to running at a constant velocity of 5.5 m/s or 19.8 km/h on grass); the number of accelerations (> 3 m/s^2^ with minimum duration of 0.5 s); the number of decelerations (< -3 m/s^2^ with minimum duration of 0.5 s) [34] were examined. Distances covered per minute (m/min) in the following categories: TD; HSR distance (> 5.5 m/s); sprint distance (> 7 m/s); HML efforts (n/min); the number of accelerations (> 3 m/s^2^; n/min) and decelerations (< -3 m/s^2^; n/min) were also observed. The mean average and peak average of each given metric per minute during match-play were obtained and analysed across all study seasons.

For each match, the effect of tactical variables were recorded utilising the Second Spectrum system (Second Spectrum, California, USA). Competitive match data was recorded using a semi-automated camera tracking system (Second Spectrum, California, USA) with a sampling frequency of 25 Hz, which has previously been installed to standardise match data collection in the EPL. The installation process, reliability and validity of Second Spectrum have previously been validated to verify the capture process and accuracy of data as reported by the FIFA Electronic Performance Tracking Systems [35]. The tactical variables examined included: possession (duration the home team had the ball divided by ball in play time) and team formation.

All examined metrics were expressed in meters, m/min and number of efforts (HML efforts only). Before calculating these values, when the individual match playing time was less than 90-minutes, distances were extrapolated to 90-minutes utilising the meters per minute calculation. All variables obtained were calculated or pre-determined in the Second Spectrum System Software. These variables have been previously utilised by soccer practitioners to longitudinally track player external load [36].

### Statistical Analysis

#### Data Sorting

Preliminary *k*-means cluster analyses were performed to identify a cut-off value of ball possession percentage and classify matches in which the study team had very low (VL) possession, low (L) possession, high (H) possession, and very high (VH) possession. The results identified cluster 1 (VL) with 35.2% of ball possession (range of 27.5–39.3%) cluster 2 (L) with 43.3% of ball possession (range of 39.4–47.1%), cluster 3 (H) with 50.9% of ball possession (range of 47.2–55.4%), and cluster 4 (VH) with 59.9% of ball possession (range of 55.5–65.7%).

### Data Analysis

Descriptive data (mean ± SD) were determined for all GPS variables of interest for the different formations (3-5-2 and 4-3-3), and possession classification (VL, L, H, VH). Homogeneity of variance was assessed via Levene’s statistic and, where violated, Welch’s adjustment was used to correct the F-ratio. Two-way (2 × 4) analysis of variance (ANOVA’s) were conducted to identify differences in physical running outputs with the different formations and possession classifications. These were conducted for all positions combined and for each specific position (centre-back, full-back, centre midfield, attacking midfield, and centre forward). Post-hoc analysis was used to identify the formation and possession classification that were significantly different to one another using either Bonferroni or Games-Howell post-hoc analyses, where equal variances were and were not assumed, respectively.

Effect size (*η*^2^) values and Cohen’s *d* values (*d*) were also reported for significant results. *η*^2^ values in the range 0–0.009 are considered insignificant effect sizes, 0.01–0.0588 as small effect sizes, 0.0589–0.1379 as medium effect sizes, and values greater than 0.1379 as large effect sizes. Cohens *d* effect size magnitudes were interpreted using the following classifications: trivial < 0.19; small 0.2–0.59; 0.6–1.19 moderate; 1.2–1.9 large; 2.0–3.9 very large; > 4.0 extremely large [37]. All significance values were accepted at p < 0.05 and all statistical procedures were conducted using JASP (version 0.18) for Macintosh.

## RESULTS

### Team

**FIG. 1 A–D:**
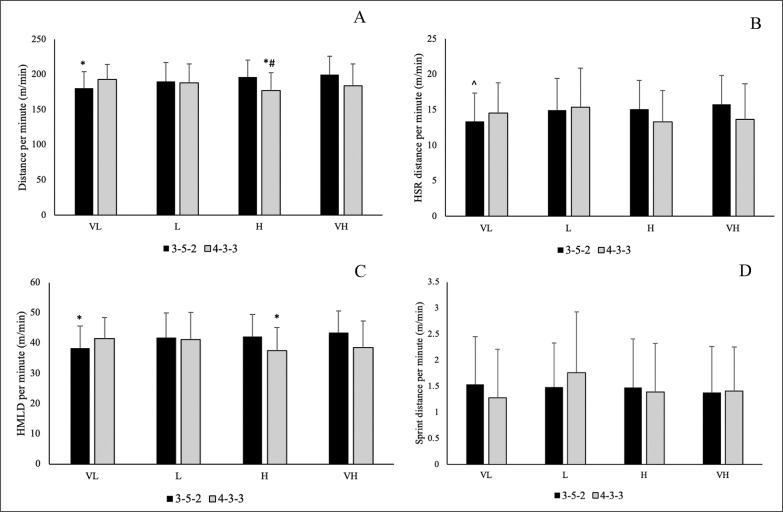
Descriptive statistics for match running demands relative to effective ball time across each possession classification and between formations (mean ± SD). A: Total distance per minute; B: High-Speed running (HSR) distance per minute; C: High Metabolic Load distance (HMLD) per minute; D: Sprint distance per minute. * significantly less than 3-5-2 L, 3-5-2 H, 3-5-2 VH; # significantly less than 4-3-3 VL; ^ significantly less than 3-5-2 L Note: VL: Very low possession percentage; L: Low possession percentage; H: High possession percentage; VH: Very high possession percentage

Descriptive statistics (mean ± standard deviation) for distances covered at different intensities for the various possession classifications and formations are presented in Figure 1. For distance covered per minute, there was a main effect for formation (p = 0.006; *η2* = 0.010), where players covered significantly more distance in a 3-5-2 formation compared to a 4-3-3 formation (p = 0.006; *d* = 0.243). There was also a significant interaction effect with formation × possession (p < 0.001; *η2* = 0.043), as players covered significantly less total distance per minute in a 3-5-2 formation when having very low possession and a 4-3-3 formation with high possession, compared to 3-5-2 formation with low, high, and very high possession (p = 0.001–0.014; *d* = 0.528–0.893). Players also covered a lower total distance per minute in a 4-3-3 formation with high possession compared to a 4-3-3 formation with very low possession (p = 0.028; *d* = 0.620).

For HSR distance per minute, there was a significant interaction effect with formation × possession (p = 0.006; *η2* = 0.018), as players covered less HSR distance per minute in a 3-5-2 formation when having very low possession compared to a 3-5-2 formation with low possession (p = 0.039; *d* = 0.361). There was a significant main effect for formation for HMLD per minute (p = 0.009; *η2* = 0.009). Where players covered significantly more HMLD per minute in a 3-5-2 formation compared to a 4-3-3-formation (p = 0.009; *d* = 0.232).

There was also a significant interaction effect with formation× possession (p < 0.001; *η2* = 0.030), where players covered less HMLD in a 3-5-2 formation with very low possession and 4-3-3 formation with high possession compared to a 3-5-2 formation with low, high, and very high possession (p = 0.001–0.027; *d* = 0.442–0.762). There were no main or interaction effects for sprint distance per minute.

**FIG. 2 A–D:**
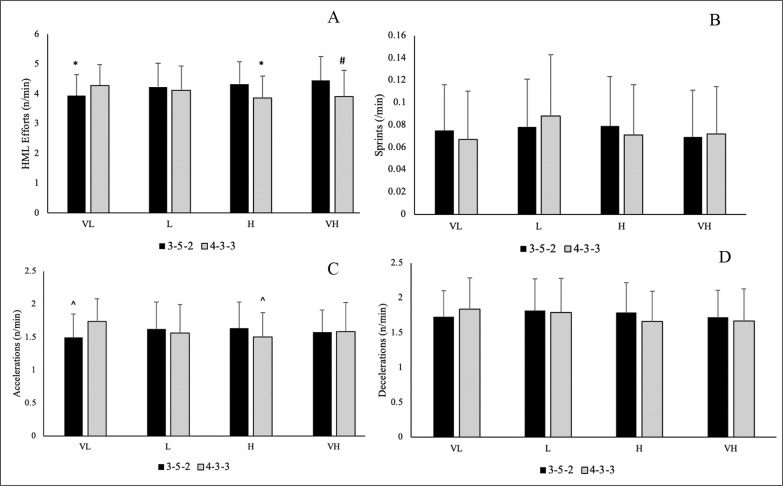
Descriptive statistics for match running demands relative to effective ball time across each possession classification and between formations (mean ± SD). A: Number of High Metabolic Load (HML) efforts per minute; B: Number of sprints per minute; C: Number of accelerations per minute; D: Number of decelerations per minute * significantly less than 3-5-2 L, 3-5-2 H, 3-5-2 VH; # significantly less than 3-5-2 VH; ^ significantly less than 4-3-3 VL Note: VL: Very low possession percentage; L: Low possession percentage; H: High possession percentage; VH: Very high possession percentage

Descriptive statistics (mean ± standard deviation) for the number of high intensity efforts for the various possession classifications and formations are presented in Figure 2. For the number of HML efforts per minute, there was a main effect for formation (p = 0.004; *η2* = 0.011), where players performed more HML efforts in a 3-5-2 formation compared to a 4-3-3 formation (p = 0.004; *d* = 0.255). There was also a significant interaction effect for formation × possession (p < 0.001; *η2* = 0.035), as players completed significantly less HML efforts per minute in a 3-5-2 formation when having very low possession and a 4-3-3 formation with high possession, compared to 3-5-2 formation with low, high, and very high possession (p = 0.002–0.013; *d* = 0.396–0.764). Players also performed less HML efforts per minute in a 4-3-3 formation with very high possession compared to a 3-5-2 formation with very high possession (p = 0.032; *d* = 0.706).

For number of accelerations per minute, there was a significant interaction effect with formation × possession (p < 0.001; *η2* = 0.025), as players covered less accelerations per minute in a 3-5-2 formation when having very low possession and in a 4-3-3 formation with high possession compared to a 4-3-3 formation with very low possession (p = 0.018–0.040; *d* = 0.601–0.622). There were no main effect of possession or formation, as well no interaction effects for number of sprints or decelerations per minute.

### Position Specific

Table 1 shows the descriptive statistics (mean ± standard deviation) for distances covered at different intensities for each position in terms of possession classification and formation. For centre-backs, there was a main effect for distance covered per minute for formation (p = 0.027; *η2* = 0.023), where this position covered more distance per minute in a 3-5-2 formation than a 4-3-3 formation (p = 0.027; *d* = 0.390). There was also a significant interaction effect for formation and possession (p = 0.001; *η2* = 0.077), as centre-backs covered less distance per minute a 4-3-3 formation with high possession compared to in a 3-5-2 formation with low, high, and very high possession (p = 0.008–0.029; *d* = 0.855–1.263).

**TABLE 1:** Match running distances for each position depending on formation and possession

	Formation	Possession	Centre Backs	Full Backs	Centre Midfield	Attacking Midfield	Centre Forward
Total Distance (m/min)	3-5-2	VL	178.12 ± 16.53	188.80 ± 17.82	181.01 ± 31.18	172.51 ± 25.24	179.27 ± 15.29
		L	188.64 ± 16.02	195.66 ± 20.81	192.72 ± 35.08	185.11 ± 34.84	189.18 ± 13.22
		H	192.44 ± 23.49	194.76 ± 27.62	208.17 ± 26.03	191.32 ± 22.94	191.81 ± 11.93
		VH	196.33 ± 19.12	188.79 ± 34.27	212.91 ± 27.28	195.12 ± 29.89	191.91 ± 16.70

	4-3-3	VL	192.32 ± 16.08	200.17 ± 18.31	200.50 ± 25.14	173.27 ± 24.32	196.21 ± 3.13
		L	180.78 ± 17.80	190.29 ± 25.09	204.01 ± 29.82	180.24 ± 28.56	184.28 ± 21.02
		H	172.52 ± 19.21^a^	172.45 ± 26.01^c^	189.89 ± 28.48	169.78 ± 26.67	175.43 ± 10.89
		VH	180.42 ± 29.08	182.83 ± 23.24	188.67 ± 35.12	182.01 ± 40.45	184.14 ± 15.43

High-Speed Running Distance (m/min)	3-5-2	VL	10.02 ± 3.02^b^	16.11 ± 3.89	14.24 ± 2.92	15.67 ± 3.59	12.18 ± 1.90
		L	11.70 ± 2.91	17.75 ± 3.96	15.52 ± 3.88	17.80 ± 5.36	14.05 ± 1.91
		H	13.13 ± 3.72	17.76 ± 5.27	15.15 ± 3.35	17.63 ± 3.74	13.97 ± 2.53
		VH	13.89 ± 3.04	17.14 ± 4.32	16.13 ± 3.90	17.60 ± 5.48	14.68 ± 3.78

	4-3-3	VL	10.08 ± 2.31	14.77 ± 2.30	16.37 ± 3.33	17.89 ± 5.22	13.95 ± 2.69
		L	10.54 ± 2.67	18.25 ± 5.58	15.56 ± 4.90	19.06 ± 5.10	14.14 ± 2.69
		H	9.60 ± 3.29^b^	14.41 ± 3.90^c^	13.27 ± 3.52	17.42 ± 3.82	12.08 ± 1.76
		VH	9.63 ± 2.66	15.78 ± 3.36	13.68 ± 3.85	18.43 ± 6.35	10.78 ± 2.74

HMLD (m/min)	3-5-2	VL	33.18 ± 5.57^a^	42.26 ± 5.23	41.82 ± 6.86	39.44 ± 7.12	33.11 ± 3.32
		L	36.86 ± 5.04	44.98 ± 5.86	45.56 ± 7.52	44.43 ± 11.1	35.45 ± 2.77
		H	38.94 ± 6.45	44.45 ± 8.03	47.09 ± 6.37	43.32 ± 6.70	35.94 ± 2.95
		VH	40.38 ± 5.45	43.35 ± 7.90	47.89 ± 4.24	43.26 ± 10.05	39.05 ± 7.88

	4-3-3	VL	35.87 ± 3.19	42.84 ± 3.65	46.31 ± 5.54	42.56 ± 10.86	37.26 ± 4.36
		L	34.31 ± 4.76	43.15 ± 9.33	46.79 ± 7.83	43.36 ± 8.97	35.48 ± 6.16
		H	32.21 ± 5.11^a^	37.84 ± 6.01	41.44 ± 7.03	40.97 ± 8.07	32.04 ± 3.25
		VH	33.04 ± 6.42	41.76 ± 5.80	40.22 ± 7.69	42.46 ± 12.87	32.18 ± 4.59

Sprint Distance (m/min)	3-5-2	VL	1.46 ± 0.91	1.85 ± 0.98	1.33 ± 0.80	1.64 ± 0.87	1.66 ± 1.15
		L	1.40 ± 0.90	1.64 ± 1.07	1.27 ± 0.65	1.68 ± 0.72	1.81 ± 0.91
		H	1.63 ± 1.12	1.22 ± 0.64	1.28 ± 0.72	1.74 ± 1.04	1.29 ± 0.88
		VH	1.43 ± 0.49	2.14 ± 1.46	0.99 ± 0.54	1.67 ± 1.14	1.01 ± 0.57

	4-3-3	VL	0.66 ± 0.54	1.63 ± 0.95	1.50 ± 1.09	1.37 ± 0.78	1.38 ± 1.15
		L	1.25 ± 0.75	2.09 ± 1.00	1.72 ± 1.16	2.28 ± 1.49	1.42 ± 1.08
		H	1.20 ± 0.94	1.40 ± 0.97	1.35 ± 0.95	1.65 ± 0.96	1.45 ± 0.69
		VH	1.18 ± 0.96	1.66 ± 0.69	1.30 ± 0.88	1.84 ± 0.67	1.10 ± 0.91

VL: Very Low Possession; L: Low Possession; H: High Possession; VH: Very High Possession. HMLD: High Metabolic Load Distance;

a : Less than 3-5-2 L, H, VH for Centre backs (p < 0.05);

b : Less than 3-5-2 H, VH for Centre backs (p < 0.05);

c : Less than 3-5-2 L for Full backs (p < 0.05)

In terms of HSR distance per minute, there was a main effect for formation (p < 0.001; *η2* = 0.079), as centre-backs covered more HSR distance per minute in a 3-5-2 formation compared with a 4-3-3 formation (p < 0.001; *d* = 0.729). There was also a significant interaction effect for formation and possession (p = 0.019; *η2* = 0.047), as centre-backs covered less HSR distance per minute in a 3-5-2 formation with very low possession and a 4-3-3 formation with high possession compared to a 3-5-2 formation with high, and very high possession (p = 0.002–0.016; *d* = 1.019–1.407).

There was a main effect for formation (p < 0.001; *η2* = 0.061) and HMLD per minute, as centre-backs covered more HMLD per minute in 3-5-2 formations than a 4-3-3 formation (p < 0.001; *d* = 0.647). There was also a significant interaction effect for formation and possession (p < 0.001; *η2* = 0.078), as centre-backs covered less HMLD per minute in a 3-5-2 formation with very low possession and a 4-3-3 formation with high possession compared to 3-5-2 formations with low, high, and very high possession (p = 0.001–0.032; *d* = 0.684–1.519). There were no main or interaction effects for sprint distance per minute for centre-backs.

Full-backs completed less TD per minute and HSR distance per minute in 4-3-3 formations with high possession compared to 3-5-2 formations with low possession (p = 0.029 and 0.009; *d* = 1.035 and 1.145, respectively). There were no main or interaction effects for HMLD or sprint distance per minute for full-backs.

There were also no main or interaction effects for TD per minute, HSR distance per minute, HMLD per minute or sprint distance per minute for centre midfielders, attacking midfielders and centre forwards.

Table 2 shows the descriptive statistics (mean ± standard deviation) for number of efforts at various intensities per minute for each position in terms of possession classification and formation. For centre-backs, there was a main effect for HML efforts per minute for formation (p = 0.006; *η2* = 0.035), where this position performed more HML efforts per minute in 3-5-2 formations than 4-3-3 formations (p = 0.006; *d* = 0.482). There was also a significant interaction effect for formation and possession (p = 0.002; *η2* = 0.071), as centre-backs performed more HML efforts per minute 3-5-2 formations with high possession compared with 4-3-3 formations with high possession and a 3-5-2 formation with very low possession (p = 0.007 and 0.004; *d* = 1.068 and 1.019, respectively).

**TABLE 2:** Number of high intensity efforts for each position depending on formation and possession

	Formation	Possession	Centre Backs	Full Backs	Centre Midfield	Attacking Midfield	Centre Forward
HML Efforts (n/min)	3-5-2	VL	3.58 ± 0.60	4.16 ± 0.44	4.32 ± 0.76	3.86 ± 0.68	3.38 ± 0.40
		L	3.93 ± 0.50	4.43 ± 0.56^a^	4.64 ± 0.82	4.32 ± 1.06	3.47 ± 0.30
		H	4.15 ± 0.64^ab^	4.37 ± 0.67	4.95 ± 0.72	4.23 ± 0.68	3.62 ± 0.31
		VH	4.19 ± 0.55	4.33 ± 0.66	5.08 ± 0.67	4.19 ± 1.02	3.84 ± 0.60

	4-3-3	VL	3.93 ± 0.40	4.55 ± 0.39	4.75 ± 0.64	4.01 ± 1.05	3.95 ± 0.26
		L	3.68 ± 0.48	4.30 ± 0.75	4.78 ± 0.79	3.98 ± 0.80	3.46 ± 0.66
		H	3.55 ± 0.49	3.84 ± 0.54	4.37 ± 0.71	3.89 ± 0.82	3.16 ± 0.31
		VH	3.61 ± 0.78	4.21 ± 0.61	4.16 ± 0.86	3.94 ± 1.16	3.27 ± 0.52

Number of Sprints (n/min)	3-5-2	VL	0.07 ± 0.04	0.09 ± 0.04	0.06 ± 0.03	0.09 ± 0.04	0.08 ± 0.04
		L	0.07 ± 0.04	0.09 ± 0.05	0.06 ± 0.03	0.10 ± 0.05	0.09 ± 0.05
		H	0.08 ± 0.04	0.06 ± 0.03	0.07 ± 0.03	0.10 ± 0.05	0.09 ± 0.06
		VH	0.07 ± 0.02	0.09 ± 0.06	0.05 ± 0.03	0.08 ± 0.07	0.06 ± 0.02

	4-3-3	VL	0.05 ± 0.04	0.09 ± 0.04	0.07 ± 0.05	0.07 ± 0.04	0.07 ± 0.04
		L	0.06 ± 0.03	0.10 ± 0.04	0.09 ± 0.05	0.12 ± 0.07	0.08 ± 0.05
		H	0.06 ± 0.03	0.08 ± 0.05	0.06 ± 0.04	0.09 ± 0.05	0.07 ± 0.04
		VH	0.05 ± 0.04	0.08 ± 0.04	0.07 ± 0.03	0.10 ± 0.04	0.08 ± 0.06

Number of Accelerations (n/min)	3-5-2	VL	1.35 ± 0.25	1.72 ± 0.26	1.38 ± 0.37	1.67 ± 0.47	1.64 ± 0.17
		L	1.58 ± 0.31^ab^	1.70 ± 0.29	1.49 ± 0.34	1.81 ± 0.70	1.73 ± 0.28
		H	1.56 ± 0.32	1.92 ± 0.33	1.45 ± 0.30	1.78 ± 0.59	1.74 ± 0.15
		VH	1.50 ± 0.12	1.68 ± 0.32	1.56 ± 0.27	1.53 ± 0.68	1.72 ± 0.19

	4-3-3	VL	1.66 ± 0.25^a^	1.87 ± 0.20	1.61 ± 0.36	1.78 ± 0.53	1.90 ± 0.23
		L	1.35 ± 0.32	1.78 ± 0.44	1.49 ± 0.40	1.68 ± 0.51	1.70 ± 0.31
		H	1.32 ± 0.25	1.55 ± 0.26	1.38 ± 0.31	1.77 ± 0.46	1.68 ± 0.25
		VH	1.37 ± 0.27	1.85 ± 0.39	1.35 ± 0.29	1.98 ± 0.57	1.68 ± 0.33

Number of Decelerations (n/min)	3-5-2	VL	1.49 ± 0.22	2.08 ± 0.25	1.78 ± 0.38	1.72 ± 0.45	1.56 ± 0.17
		L	1.57 ± 0.30^a^	2.15 ± 0.36^a^	1.86 ± 0.39	1.91 ± 0.70	1.69 ± 0.23
		H	1.59 ± 0.28	2.21 ± 0.40	1.87 ± 0.34	1.77 ± 0.64	1.78 ± 0.26
		VH	1.49 ± 0.16	2.05 ± 0.26	1.78 ± 0.20	1.67 ± 0.75	1.76 ± 0.27

	4-3-3	VL	1.52 ± 0.32	2.20 ± 0.27	1.87 ± 0.26	1.91 ± 0.75	1.70 ± 0.30
		L	1.44 ± 0.28	2.03 ± 0.41	1.94 ± 0.50	1.89 ± 0.56	1.69 ± 0.37
		H	1.35 ± 0.29	1.81 ± 0.39	1.72 ± 0.43	1.85 ± 0.51	1.59 ± 0.23
		VH	1.35 ± 0.32	2.03 ± 0.47	1.57 ± 0.28	1.93 ± 0.62	1.62 ± 0.31

VL: Very Low Possession; L: Low Possession; H: High Possession; VH: Very High Possession; HML: High Metabolic Load.

a : More than 4-3-3 H (p < 0.05);

b : More than 3-5-2 VL (p < 0.05).

Considering the number of sprints per minute, there was a main effect for formation (p = 0.004; *η2* = 0.044), as centre-backs performed more sprints per minute in a 3-5-2 formation than a 4-3-3 formation (p = 0.004; *d* = 0.513).

In terms of number of accelerations per minute, there was a significant interaction effect for formation and possession (p < 0.001; *η2* = 0.103), as centre-backs performed more accelerations per minute in a 3-5-2 formation with low possession and a 4-3-3 formation with very low possession compared to a 4-3-3 formation with high possession (p = 0.002 and 0.045; *d* = 0.947 and 1.212). Centre-backs also completed more accelerations per minute in 3-5-2 formations with low possession compared to 3-5-2 formations with very low possession (p = 0.002; *d* = 0.840).

There was a main effect for formation (p = 0.011; *η2* = 0.032) and the number of decelerations per minute, as centre-backs covered more decelerations per minute in 3-5-2 formations than 4-3-3 formations (p = 0.011; *d* = 0.447). Centre-backs also completed more decelerations per minute in a 3-5-2 formation with low possession compared to a 4-3-3 formation with high possession (p = 0.019; *d* = 0.813).

Full-backs completed more HML efforts and decelerations per minute in a 3-5-2 formation with low possession compared to a 4-3-3 formation with high possession (p = 0.020 and 0.041; *d* = 1.072 and 1.003, respectively).

There were no main or interaction effects for HML efforts per minute, sprints per minute, accelerations per minute or decelerations per minute for centre midfielders, attacking midfielders and centre forwards.

## DISCUSSION

The aim of this study was to examine the effect of possession, team formation and playing position on physical match performance variables across two consecutive seasons of elite soccer match-play. The main observations from this study were that a significant main effect for formation on TD covered per minute was observed. Specifically, players covered significantly greater TD in a 3-5-2 formation compared to a 4-3-3 formation. Furthermore, there was a significant interaction effect between formation and possession on TD covered per minute. Particularly, players covered significantly lower TD per minute in a 3-5-2 formation when having very low possession and in a 4-3-3 formation with high possession, compared to a 3-5-2 formation with low, high, and very high possession. Additionally, players covered a lower TD per minute in a 4-3-3 formation with high possession compared to a 4-3-3 formation with very low possession. These findings suggests that the interaction between possession and formation can influence the distance covered by players, with different formations being more or less effective depending on the possession status of the team.

### Team

For HSR distance per minute, there was a significant interaction effect between formation and possession. Specifically, players covered less HSR distance per minute in a 3-5-2 formation when having very low possession compared to a 3-5-2 formation with low possession. This highlights that possession interacts with team formation and affects the quantity of HSR performed by the team. Potentially, this may partly be explained by the in-possession tactical strategy adopted by the study team when choosing a 3-5-2 formation. The 3-5-2 formation can arguably be classified as a defensive formation and is often implemented against higher quality opponents and thus may reflect the very low possession observed in the current study. While also explaining less HSR distance per minute as this metric is often witnessed while undertaking attacking phases of play which were less evident in the study team during matches of low and very low possession. A recent study [19] that examined three elite soccer teams found that one of the analysed teams (Team 1) that had a more defensive style (4-4-2 formation) expended more time in defending actions and initiating more direct attacks. The same study [19] analysed two other teams that employed 4-4-3 formations in which one team employed an indirect style of play (Team 2) while the third team employed intricate attacks and effective counter-attacks (Team 3). While Team 2 employed complex attacks using short, explosive movements and a higher number of passes between thus decreasing the distances covered, Team 3 showed a greater number of counter-attacks and therefore covered greater distances at higher speeds. As an example, distance covered at > 14 km/h was higher in Team 1 compared with Team 2. Despite the different formation of Team 2 when compared with Team 1 of the previous study [19], the 3-5-2 formation seemed to have a similar defensive approach that may help explain the results, leading to the relevance of the tactical strategy adopted.

There were no main or interaction effects observed for sprint distance per minute. This may imply that neither the formation nor possession had a significant impact on sprint distance covered by players during match-play. Nonetheless, a different scenario occurred where a 4-3-3 formation team produced a greater number of counter-attacks that led to higher sprint distances, while another team employing the same 4-3-3 formation performed more complex attacks, with shorter movements and a higher number of passes that decreased sprint distances [17, 19, 33].

Furthermore, there was a significant interaction effect between formation and possession for the number of HML efforts per minute. This interaction effect revealed that in a 3-5-2 formation, the number of HML efforts per minute decreased significantly when possession was very low compared to a 3-5-2 formation with low, high, and very high possession. Similarly, in a 4-3-3 formation, the number of HML efforts per minute decreased significantly when the possession was high compared to a 3-5-2 formation with low, high, and very high possession. Additionally, players performed fewer HML efforts per minute in a 4-3-3 formation with very high possession compared to a 3-5-2 formation with very high possession. This may partly be explained by a defensive tactical strategy that does not promote a ‘high-press’ and maintains a compact defensive shape and thus reduces HML efforts even with very low possession. Although, lower HML efforts per minute in a 4-3-3 formation with high possession may also suggest a ‘building’ approach when in possession compared to a more direct strategy that would require running in behind. For instance, a previous study compared the effects of high and low percentage ball possession of EPL teams (no formation analysis) on physical and technical profiles during elite soccer match-play and concluded that ball possession percentage does not influence TD. Although, ball possession does influence HSR efforts with and without the ball and some technical elements of performance [10, 14, 38].

A further identical study with data from FIFA World Cup reported that ball possession does not influence the activity patterns of international matches although high possession teams spend more time in offensive areas of the pitch (again no formation was considered for analysis) [39]. Moreover, other research [40] showed that HSR with ball possession in offensive, traditional formations (4-3-3 and 4-4-2) were ~30 to 40% higher than defensive formations (4-5-1). The same study reported that around 20% more HSR distance was covered without possession in defensive versus offensive, traditional formations [40], which again reinforces the importance of considering the tactical strategy.

In terms of the number of accelerations per minute, there was a significant interaction effect between formation and possession. Specifically, in a 4-3-3 formation, players performed fewer accelerations per minute with high possession compared to a 4-3-3 formation with very low possession. Therefore, it may be speculated that when selecting a 4-3-3 formation, that is often considered an attacking formation, and attempting to maintain high possession, this tactical combination lowers explosive acceleration actions performed by all positions, as these are frequently associated with defensive ‘pressing’ actions. This potentially suggests that the combination of formation and possession can have an impact on the number of accelerations performed by players regardless of playing position.

### Position Specific

When analysing physical match performance and playing position based on possession and formation, centre-backs revealed a main effect for TD covered per minute in relation to formation. Specifically, centre-backs covered more TD per minute in a 3-5-2 formation than a 4-3-3 formation. Centre-backs also covered less TD per minute a 4-3-3 formation with high possession compared to a 3-5-2 formation with low, high, and very high possession. Regarding HSR distance per minute, centre-backs showed a main effect for formation, indicating that this position covered more HSR distance per minute in a 3-5-2 formation compared to a 4-3-3 formation. The practical significance of this effect was moderately small. Although, when considering the previous study that analysed three elite Spanish teams with differing formations and playing strategies, contrasting results emerge as centre-backs were reported to cover higher distances in teams with 4-4-2 formations and a defensive style, however that was not evident [19].

Similarly, a significant interaction effect was observed between formation and possession for HSR distance per minute for centrebacks. In this case, centre-backs covered less HSR distance per minute in a 3-5-2 formation with very low possession and a 4-3-3 formation with high possession compared to a 3-5-2 formation with high and very high possession. These effect sizes were moderately large, indicating a considerable practical significance that sustains the relevance of contextualising match physical demands by also analysing ball possession [39].

Moreover, a significant interaction effect between formation and possession was found for HMLD per minute in centre-backs. Specifically, centre-backs covered less HMLD per minute in a 3-5-2 formation with very low possession and a 4-3-3 formation with high possession compared to 3-5-2 formations with low, high, and very high possession. These effect sizes suggested moderately large practical significance. A study conducted with Chinese Premier League players also reported that centre-backs covered more high-intensity (5.5–7 m/s) and sprint (> 7 m / s) distance in the high possession teams, although no formation analysis was considered [40], which also leads to the importance of ball possession. Full-backs covered less TD per minute and HSR distance per minute in 4-3-3 formations with high possession compared to 3-5-2 formations with low possession. These differences were statistically significant and demonstrated moderately large effect sizes.

In terms of accelerations per minute, there was a significant interaction effect between formation and possession for centre-backs. Centre-backs performed more accelerations per minute in a 3-5-2 formation with low possession and a 4-3-3 formation with very low possession compared to 4-3-3 formations with high possession. Additionally, centre-backs completed more accelerations per minute in 3-5-2 formations with low possession compared to 3-5-2 formations with very low possession. This indicates that time in possession, in combination with formation, has a significant effect on the number of acceleration efforts made by centre-backs as this defensive position will potentially be required to produce more out of possession ‘pressing’ actions or perform more 1 v 1 moments. The effect size here also suggests a large influence on physical performance for this position.

For the number of decelerations per minute, there was a significant main effect for formation. Centre-backs covered more decelerations per minute in 3-5-2 formations compared to 4-3-3 formations. This suggests that the tactical setup also influences the frequency of decelerations for centre-backs. The effect size indicates a small to moderate influence of formation on decelerations for centre-backs. Additionally, centre-backs completed more decelerations per minute in a 3-5-2 formation with low possession compared to a 4-3-3 formation with high possession. Furthermore, full-backs exhibited higher numbers of HML efforts and decelerations per minute in a 3-5-2 formation with low possession compared to a 4-3-3 formation with high possession. This not only indicates that the tactical setup affects these explosive, out-of-possession performance metrics for full-backs but also suggests that possession plays a significant role in the physical demands for this position.

Furthermore, the 3-5-2 formation may gain an advantage where the wing-back role acting as an external defender will have longer runners to cover due to the multi-faceted role this position plays, including the role of wide midfielders. According to previous research, there may be three justifications: 1) when the team with higher possession attacks, three lines of players move together deep into the opponents’ half which produces greater distances for centre-backs and wing-backs to cover [40]; 2) when the team with higher possession defends, wing-backs and centre-backs will need to sprint to mark opponent players or chase the ball until the ball is intercepted, resulting in fast counter-attacks [40]; and 3) teams with low ball possession have to choose a counter-attack strategy when facing high quality opponents as low possession teams have fewer chances of achieving penetrative passes, thus have to exploit any weaknesses in the opponents’ defence and effectively take advantage of an imbalanced defensive line [41, 42]. However, no main or interaction effects were observed for HMLD or sprint distance per minute for full-backs. Lastly, no main or interaction effects were found for any of the distance measures (distance per minute, HSR distance per minute, HMLD per minute, and sprint distance per minute) for centre midfielders, attacking midfielders, and centre forwards.

In summary, the results highlight the importance of both formation and possession in relation to the distances covered at different intensities, specifically for centre-backs and full-backs. These findings suggest that tactical and strategic factors, such as formation and possession, influence the physical demands placed on players in different positions.

### Practical implications, limitations, and future research directions

Overall, these team findings highlight the importance of considering both the formation and possession when analysing the intensity of match efforts. Specifically, the present results suggest that different team formation and possession may lead to variations in the number of high-intensity efforts and accelerations performed. These findings may have practical implications for coaches and players in terms of optimising tactical game strategies (formation, in-possession, outof-possession and transitional approaches) and player performance based on formation and possession.

Several limitations should be noted when interpretating the findings of this study. The current data are reflective of the methods and practices of a single elite soccer club, the positional match running performance and variations resulting from possession classifications and team formation. Also, this study did not examine the outcome of effective playing time on locomotor performance [43]. In addition, other contextual factors such as opponent level [44], match location [20], match outcome [45] and match half were not considered. For example, when playing against top-level teams, players covered greater distance of high-speed running and sprinting [46]. In the same line, other study showed that more decelerations were performed against top-level than middle-and bottom-level opponents [44]. Regarding match location, a recent study showed that more acceleration and decelerations were performed when playing against home than away matches [20]. Considering the outcomes, previous research showed that winning increased external load than a draw or loss [45]. Moreover, fatigue is another variable that take a role in all results and considered the inclusion criteria adopted for this study, it is possible that some players could be fresher than others through the season or within the match (e.g. first half and second half) which could influence the results. For that reason, the inclusion of some physiological (heart rate), psychophysiological (rating of perceived exertion), or a wellness (through a questionnaire, e.g., Hooper Index) variable is warranted in future research. Finally, the context of the game is also other variables that can influence results. For instance, the matches of the season can decide if the team is regulated to a lower division or if it can participate in a European competition in the next season. Thus, future research should also include such variable in the analysis.

Consequently, the results should only be generalised to similar cohorts, level of competition, and tactical approaches. Thus, future studies should be conducted to compare current findings utilising larger sample sizes with various team formations and possession time.

## CONCLUSIONS

In conclusion, the findings of this study provide insights into the influence of formation and possession on the physical performance of soccer players. The results indicate that the choice of formation and possession can have a significant impact on TD covered, HSR distance, and HMLD per minute of elite soccer teams. These findings suggest that coaches and teams can tailor tactical strategies and formations based on possession classifications to optimise player match-play performance.

Furthermore, the study revealed that the number of high intensity efforts per minute was influenced by formation, with players performing more high intensity efforts in a 3-5-2 formation compared to a 4-3-3 formation. Additionally, the interaction between formation and possession affected the number of high intensity efforts performed, indicating that different formations and possession classifications can influence the physical demands placed on players.

In addition, the results highlight the importance of both formation and possession in relation to the distances covered at different intensities, specifically for centre-backs and full-backs in the two formations analysed. These findings suggest that both formation and possession, influence the physical demands placed on players in different positions.

## Conflict of interest

The authors declare no conflict of interest.
